# The biological function of consciousness

**DOI:** 10.3389/fpsyg.2014.00697

**Published:** 2014-08-05

**Authors:** Brian Earl

**Affiliations:** Independent Researcher, Formerly Affiliated with the School of Psychological Sciences, Monash UniversityMelbourne, Australia

**Keywords:** behavioral flexibility, components of experience, flexible response mechanism, function of consciousness, qualia array

## Abstract

This research is an investigation of whether consciousness—one's ongoing experience—influences one's behavior and, if so, how. Analysis of the components, structure, properties, and temporal sequences of consciousness has established that, (1) contrary to one's intuitive understanding, consciousness does not have an active, executive role in determining behavior; (2) consciousness does have a biological function; and (3) consciousness is solely information in various forms. Consciousness is associated with a *flexible response mechanism* (FRM) for decision-making, planning, and generally responding in nonautomatic ways. The FRM generates responses by manipulating information and, to function effectively, its data input must be restricted to task-relevant information. The properties of consciousness correspond to the various input requirements of the FRM; and when important information is missing from consciousness, functions of the FRM are adversely affected; both of which indicate that consciousness is the input data to the FRM. Qualitative and quantitative information (shape, size, location, etc.) are incorporated into the input data by a qualia array of colors, sounds, and so on, which makes the input conscious. This view of the biological function of consciousness provides an explanation why we have experiences; why we have emotional and other feelings, and why their loss is associated with poor decision-making; why blindsight patients do not spontaneously initiate responses to events in their blind field; why counter-habitual actions are only possible when the intended action is in mind; and the reason for inattentional blindness.

## 1. Introduction

Consciousness science has been the subject of considerable research effort in recent decades, and this has led to the creation of very many theories about consciousness, but none has broad acceptance within the scientific community (Pereira et al., 2010; Katz, 2013). Katz commented that the profusion of theoretical approaches suggests there is a profound problem in this domain. A central problem in consciousness science is that we still do not know the biological function of consciousness^1^ —we do not know why we have experiences (Bayne, 2009; de Gardelle and Kouider, 2009; Seth, 2009). When the function of consciousness is known, that knowledge is likely to have a significant effect on our understanding of consciousness, and on future directions in research.

Of the very many theories of consciousness proposed in recent decades, most attempt to explain how consciousness arises. Some examples are: higher-order thought (Rosenthal, 1986, 2000), integrated information (Tononi et al., 1998; Tononi, 2004), neural correlates of consciousness (Crick and Koch, 1990; Koch, 2004), re-entrant interaction (Edelman, 1992, 2003), quantum coherence (Hameroff, 1994; Hameroff and Penrose, 1996), and sensorimotor (O'Regan and Noë, 2001) theories. All of these theories, and many others, are concerned primarily with how consciousness arises, and only secondarily, if at all, with the biological function of consciousness.

The other main category of consciousness theories is those that describe how consciousness and its associated neural processes interact with other systems in the brain. Examples are global workspace theory (Baars, 1988, 1997), and supramodular interaction theory (Morsella, 2005). Each of these two theories also includes a statement of how consciousness arises.

Several of these theories refer to the biological function of consciousness, with statements ranging from the general comment that consciousness is adaptive, to statements that consciousness functions as a form of workspace with input from and output to various kinds of unconscious processing (Baars, 1997), or that its function is to produce a single representation and make it available to the parts of the brain that choose between different plans for action (Crick and Koch, 1998). These and many other theories have proposed a similar function for consciousness to the present paper; to contribute to responding more flexibly when automatic actions are unsuitable. What is new about the approach presented here is that I have examined the properties of consciousness and concluded from them that consciousness can only have biological value as input to a mechanism, or mechanisms, that determine behavior.

Consciousness can only have biological value if it benefits the organism by changing its behavior. In general, an evolved property of an organism can be adaptive as a result of changes to its body, or its behavior, or both its body and its behavior, that enhance the organism's ability to survive, reproduce, and perpetuate its genetic material through subsequent generations. Such changes may assist in the avoidance of predators, getting food, digestion, healing injuries, sexual function, and so on. The evolution of conscious neural structures^2^ did not involve physical changes outside of the nervous system that could confer physical advantages such as improved camouflage, quicker movement, or improved organ function. Therefore, if consciousness is adaptive, its biological value must result from allowing improved behavioral choices for its possessors. Hence, consciousness can only have adaptive value and a biological function by virtue of its being able to influence behavior.

**Figure 1:**
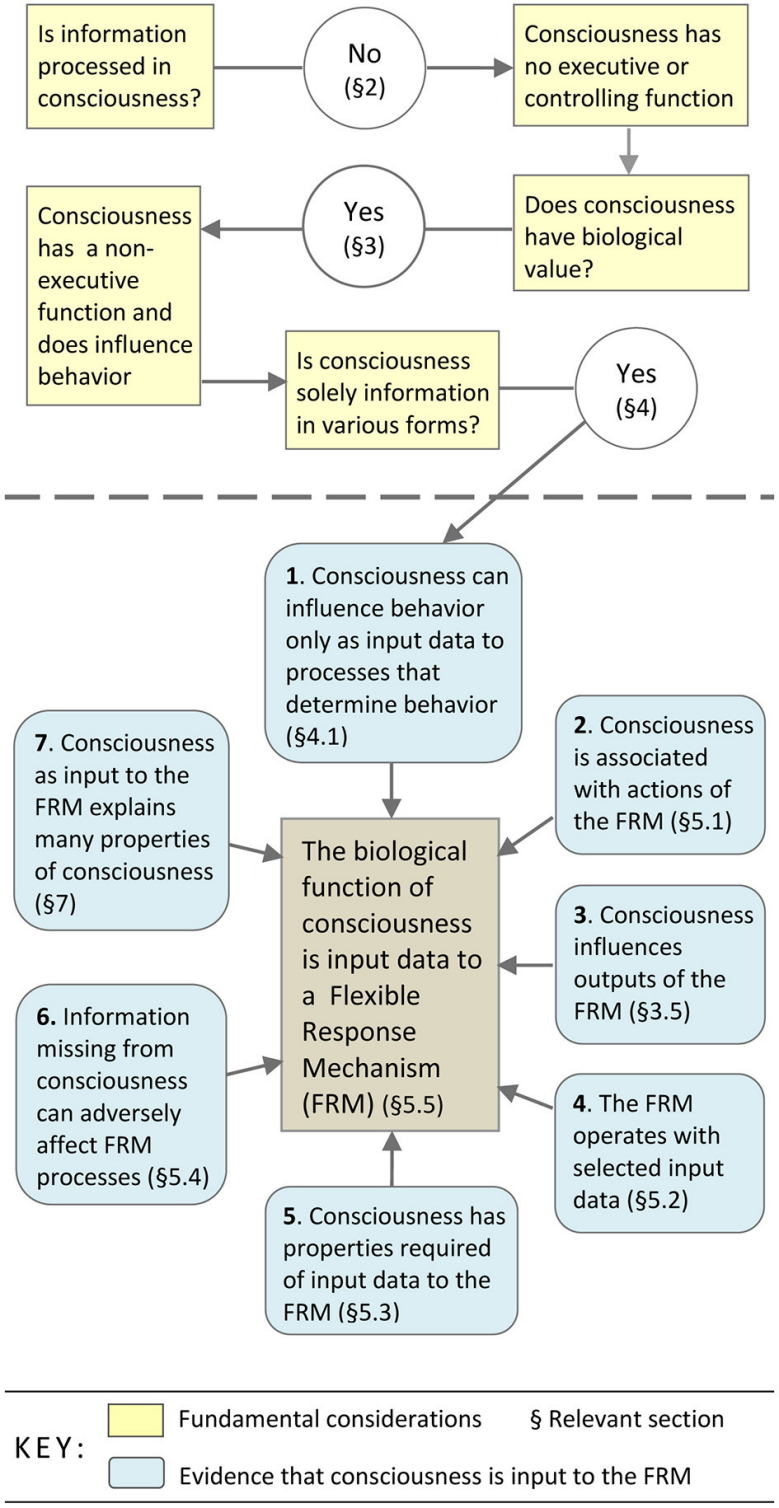
**Establishing that consciousness is input to a mechanism which generates thoughts, intentional actions, plans, decisions, and so on**.

The purpose of this research was to establish the biological function of consciousness—the actual role of consciousness in the selection and control of certain behaviors—and then to demonstrate that this biological function leads to an explanation for the known properties of consciousness. The research begins with consideration of three topics: the evidence that one experiences no mental^3^ processes^4^, the evidence that consciousness is adaptive, and the evidence that consciousness is solely information^5^. The logical sequence of this research, and the principal lines of evidence and conclusions are represented in the flowchart (**Figure 1**).

The research has two parts. The first part, shown above the broken line in the flowchart, is concerned with establishing that consciousness has a biological function, and that its function must consist in being the data input to processes for determining behavior. The second part of the research, represented below the broken line in the flowchart, is a collection of evidence pointing to consciousness being the input to a mechanism for manipulating data to generate responses in situations where automatic programs are likely to produce less than optimal behavior. This mechanism, the *flexible response mechanism* (FRM), is the source of thoughts, intentional actions, decisions, plans, daydreams, and so on.

## 2. Evidence that consciousness has no executive function

Some of the many published claims that consciousness incorporates no processing at all, or is not involved in particular kinds of processing, are listed in Section 2.1. In Section 2.2, I provide evidence from introspection and experiments that demonstrates, in relation to the principal mental activities, that there is no processing of information in consciousness.

### 2.1. Published claims that consciousness includes no mental processes

Lashley (1958, p. 4) wrote that “*No activity of mind is ever conscious*” (emphasis in original). His examples were that we do not experience how our perceptions are created, or how sentences are produced. He also wrote that acts of will, decisions, logic, and the formation and manipulation of abstractions, are all nonconscious neural processes.

Miller (1962) also made an early contribution to arguments that none of the processes of the conscious mind are experienced. He wrote that thinking is a preconscious process, for example when one recalls something from memory “consciousness gives no clue as to where the answer comes from; the processes that produce it are unconscious. It is the *result* of thinking, not the process of thinking, that appears spontaneously in consciousness” (p. 72, emphasis in original). Miller wrote that processes leading to perception are complex, and we have no awareness of them; perception is an unconscious process. “What is true of thinking and of perceiving is true in general. We can state it as a general rule: No activity of mind is ever conscious” (p. 72).

Nisbett and Wilson (1977) were the first to provide evidence, based on a review of published experimental work, that we never really know why we do the things we do, why we choose the things we choose, or why we prefer the things we prefer. When people are questioned about these matters, both the questioner and the questioned are ordinarily unaware that the answers given are necessarily fabrications. The conclusions of Nisbett and Wilson were supported by the research of Johansson et al. (2005, 2006).

In a now classic study, Velmans (1991) examined information from a variety of sources in search of evidence of processing in consciousness, and found none. After reviewing the relevant literature, and other considerations, Velmans reported that:

- Analysis and selection of information for entry to consciousness is unconscious—one is not aware of any of this analysis as it happens.
- Control of attention is unconscious—consciousness cannot select what is to enter itself.
- Learning and memory are unconscious processes—processes that create and retrieve memories are not accessible to introspection.
- Planning—for example, formulating ideas and translating them into a suitable form for speech are not conscious—one is only aware of exactly what one is going to say after one has said it.
- Creativity is not a conscious process.
- Organizing our responses is unconscious—we respond to a skin stimulus long before it has entered consciousness.
- Determining priorities is unconscious—what is important at any given moment requires continuous updating in a constantly changing world, and we are not aware of this occurring.
- Production of voluntary responses is unconscious—there is no awareness of any of the processing needed to execute a response.

Velmans concluded that data for entry into experience are preselected unconsciously; there was no direct evidence of data processing in consciousness—no choices or control of behavior occur in consciousness; and, post-conscious data processing is entirely unconscious—behavior that follows on from events in consciousness is under automatic control.

Smith (1999) stated that it is logically impossible to consciously choose one's next word or one's next experience, because they would have to be conscious already. Therefore, they are not consciously determined and must be generated by processes in the brain of which one is not conscious. And, when trying to think of the next word to write, one is occasionally conscious of a “waiting period for the next word that will appear… an *unconscious* ‘incubation’ process takes place ending when the next word appears” (Smith, 1999, p. 426). And, one is not conscious of the computations done by the brain, but only of its decisions or plans; Smith wrote that consciousness has no computational powers.

Edelman and Tononi (2000, p. 58) wrote, “when we consciously add two numbers together, it seems that we simply pass a request to our brain, the brain carries it out, and the answer is delivered…. When we search for an item in memory, we formulate the question in our consciousness. Unbeknownst to us, the brain seems to search for a while, and suddenly the response is made available to consciousness.”

Dehaene and Naccache (2001, p. 16) wrote that “we can never be conscious of the inner workings of our cerebral processes, but only of their outputs. A classical example is syntax: we can become conscious that a sentence is not grammatical, but we have no introspection on the inner workings of the syntactical apparatus that underlies this judgment.”

Wegner (2002) wrote that creative insights are unexpected and appear to be involuntary; adding two numbers occurs unconsciously; highly skilled actions happen without involving consciousness (driving a car, playing a musical instrument). He stated that “unconscious and inscrutable mechanisms create both conscious thought about action and the action, and also produce the sense of will we experience by perceiving the thought as the cause of the action” (p. 98).

Pockett (2004) wrote that problem solving is unconscious—only the solution is presented to the conscious mind, and “the creator tends to have the eerie feeling that ‘It wasn’t me who did it”' (p. 31); “I know what I think only when I see what I write” (p. 31); and that the experience of volition is not necessary for the performance of actions that would normally be considered volitional.

Umiltá(2007, p. 328) wrote that “we are never conscious of the inner workings of our cognitive processes, but only of their outputs.”

In summary, according to these researchers: The processes leading to perception are nonconscious^6^ (Lashley, 1958; Miller, 1962). Control of attention, and analysis and selection of information for entry to consciousness are nonconscious (Velmans, 1991; Smith, 1999). Intentional actions are under nonconscious control (Velmans, 1991; Wegner, 2002). Choices and decisions are nonconscious processes (Lashley, 1958; Nisbett and Wilson, 1977; Velmans, 1991; Smith, 1999; Johansson et al., 2005, 2006). Thoughts are determined nonconsciously (Miller, 1962; Velmans, 1991; Smith, 1999; Wegner, 2002; Pockett, 2004). Before speaking or writing, one may know what one is going to speak or write about, because the thought has already been nonconsciously determined, but the selection and articulation of the words are nonconscious processes (Lashley, 1958; Smith, 1999; Velmans, 2002; Libet, 2004; Pockett, 2004). Problem solving and creativity are nonconscious (Velmans, 1991; Wegner, 2002; Pockett, 2004). Skilled actions are nonconsciously controlled (Wegner, 2002). Numerical addition is a nonconscious process (Edelman and Tononi, 2000; Wegner, 2002). Memory storage and recall are nonconscious processes (Miller, 1962; Velmans, 1991; Edelman and Tononi, 2000). Logic, and the formation and manipulation of abstractions, are nonconscious processes (Lashley, 1958). There is no evidence of any data^7^ processing in consciousness (Miller, 1962; Velmans, 1991; Smith, 1999; Dehaene and Naccache, 2001; Umiltá, 2007). This is not a complete list of such claims, but I hope it is sufficient to establish that many researchers have come to the conclusion either that one experiences no mental processes, or that at least some of what are regarded, according to commonsense, as mental processing functions of consciousness are, in reality, never experienced.

In what follows, I examine a number of these claims in more detail. My aim is to present evidence that supports these claims, and refutes other claims, which continue to appear in scientific and philosophical publications, that our thoughts or feelings choose, initiate or control our actions, or that one can observe every stage in a “conscious” choice or decision.

### 2.2. Introspective and experimental evidence that one experiences no mental processes

Rosenthal (1986, p. 356) commented that “relative to what we now know about other natural phenomena, we still have strikingly scant understanding of the nature of the mental. So introspection looms large as a source of information, just as sense perception was a more central source of knowledge about physical reality before the flourishing of the relevant systematic sciences.” For those who have developed the necessary skills, introspection can be an important source of information about consciousness, and such information is especially valuable when there is a convergence between subjective and objective measures (Jack and Shallice, 2001; Jack, 2013).

In the following subsections, I describe evidence from both introspection and experiments and, as will be apparent, these two lines of evidence are mutually confirmatory whenever both are available for similar situations. This evidence supports the view of the researchers listed in the previous section who stated that one does not experience the actual mental process during any mental activity, and it demonstrates that consciousness cannot have an executive role. One may experience the starting conditions, stages during processing, or the final outcome of mental processes, but there are always gaps in one's experience, and one never experiences the actual data manipulation that generates the outcome.

#### 2.2.1. Intentionally initiating an action is a nonconscious process

When I decide to pick up a cup and do so, I may believe that my thought initiates my action, but what I observe is I have the thought of picking up the cup and then reach out and take the cup. I do not experience the information manipulations that must occur to initiate my action, and I have no evidence that my action is consciously initiated. One tends to assume one's intentional actions are consciously initiated, but as Wegner and Wheatley (1999) reported, we may perceive our actions as consciously caused if the thought occurs before the act and is consistent with the act, and there are no other likely causes.

Libet and coworkers (Libet et al., 1983; Libet, 1996) described a series of experiments in which subjects were instructed to move a finger at a moment of their own choosing, and to note the time of awareness of “wanting,” “urge,” “intention,” or “decision” to move. Libet and coworkers recorded the time of intention to move and the time of onset of the related readiness potential (RP), a scalp-recorded negative potential, which was known to precede physical actions. They found that the RP preceded the conscious intention to act by about 350 ms. They wrote that, “the brain evidently ‘decides’ to initiate or, at the least, prepare to initiate the act at a time before there is any reportable subjective awareness that such a decision has taken place. It is concluded that cerebral initiation even of a spontaneous voluntary act, of the kind studied here, can and usually does begin *unconsciously*” (p. 640, emphasis in original). Keller and Heckhausen (1990) replicated the experiment of Libet and co-workers and confirmed their conclusions.

Haggard and Eimer (1999) repeated the Libet experiments but, in some trials, they allowed free choice which index finger to move. They also recorded the lateral readiness potential (LRP), which is a measure of the difference between levels of premotor activation on the active and inactive sides. Their data showed that the LRP was a more appropriate observation than the RP, and that the LRP preceded the intention to move, but by less time. They claimed that the experienced intention to move reflected neural events associated with implementation of the movement. Haggard (2005) wrote that the frontal and parietal lobes prepare motor actions and produce the experience of intention.

Trevena and Miller (2002) measured RPs and LRPs, and employed statistical methods to analyze their measurements. They found that LRP is the relevant indicator, and that some perceived decisions occurred before the LRP, though on average they were after the LRP. They concluded that “inferring the direction of mind-body causation on the basis of temporal discrepancy alone is complicated by the difficulty of precisely timing both neural onsets and subjective experiences” (p. 132).

Banks and Pockett (2007) reviewed all these studies, and other relevant work, and concluded that the evidence appeared to support the view that the decision to act is prepared nonconsciously before the conscious decision. Thus, the evidence from introspection and experiments supports the view that consciousness does not cause the action, but merely indicates what the action will be (Wegner and Bargh, 1998; Pockett, 2006).

Evidence from experiments that involve the subjective timing of decisions always has an element of inconclusiveness because of the difficulty of determining the exact moment of the decision. However, the conclusion from them that actions are always initiated nonconsciously is supported by the results of Hansen and Skavensky (1985), discussed below, and it is supported by introspective observations, and from the fact that a decision when to act is similar in character to other decisions for which there is evidence they are nonconsciously determined.

#### 2.2.2. Intentional control of actions is a nonconscious process

When I slowly and attentively reach out and pick up a book, I may believe my movements are consciously controlled, but what I experience is an intention to control the movements plus consciously monitoring my arm movements (Smith, 1999). There is no experience of consciously controlling the movement. For example, if I see my hand is reaching toward the wrong book my hand moves to the correct location, but the processes that adjust its movement are unknown to me; control of the motor systems is not accessible to consciousness.

When one strikes a nail with a hammer, one pays close attention to the task and might think the movement of the hammer is under conscious control. However, Hansen and Skavensky (1985) reported evidence that the hammer is controlled nonconsciously. They described an experiment in which they used a hammer to hit a briefly illuminated point of light arranged to occur somewhere on a line from left to right in front of them. At the same time, their saccadic eye movements were recorded. During saccades, they were not consciously aware of seeing the light but they swung the hammer in response to the light, and their hits were as fast and accurate as when they reported they had seen the light. Since their speed and accuracy were the same, the mechanism would appear to have been the same whether or not they were conscious of the light. The initiation and control of the hammer movement were both nonconscious processes.

Correct adjustment of hand movement toward a target that has changed position can occur under conditions where participants are not consciously aware the target has moved, and therefore their hand movement could not be under conscious control (Bridgeman et al., 1979; Goodale et al., 1986; Pélisson et al., 1986; Prablanc and Martin, 1992). Intentional movement toward a target object is corrected within 100–120 ms after hand movement begins, or after the target has changed position (Smith and Bowen, 1980; Soechting and Lacquaniti, 1983; Zelaznik et al., 1983; Castiello et al., 1991; Prablanc and Martin, 1992), but this is less than the time taken to experience movement of the hand or target, which is about 150–300 ms for most adults at ordinary stimulus intensities (Libet et al., 1964, 1991; Libet, 1965, 1985; Meador et al., 1996; Ray et al., 1998, 1999). Therefore, these intentional actions must be nonconsciously controlled.

Nonconscious visual systems can accurately control intentional behavior even when the conscious visual representation is in error, because behavior is controlled on the basis of nonconscious representations that are generally less prone to error than our perceptual representations (Bridgeman et al., 1997; Rossetti, 1998; Bridgeman, 2002; Umiltá, 2007).

The vast majority of experiments investigating the control of behavior related to whether conscious or nonconscious *visual* information was employed to control behavior, and they consistently found that control is nonconscious. However, Paillard et al. (1983) showed that unperceived *tactile* information could also control behavior. They studied a person who had suffered neurological damage that resulted in the total loss of experienced sensation on her right side. They described an experiment in which the woman was blindfolded and then touched at points on her right hand. She had no conscious awareness of the contact, but when asked to touch the point of contact with her left hand was able to do so without difficulty. The woman's action in touching the point of contact was a voluntary movement, which ordinarily would be assumed to be under conscious control. But the fact that she was unable to experience the contact showed that her movement was controlled nonconsciously. Since she had no difficulty using her nonconscious tactile information for controlling her movements it is likely that she was using the same system as she used before her injury (see Rossetti, 1998, for evidence on tactile and proprioceptive data for action).

It seems that action control is always nonconscious, whatever the data source. Evidence that consciousness does not directly control actions has been reviewed (for example, Rossetti, 1998; Rossetti and Pisella, 2002; Goodale and Humphrey, 2005; Umiltá, 2007).

#### 2.2.3. Choices and decisions are nonconscious processes

As an example of a “conscious” decision followed by action, I am engrossed in a particularly interesting article when a sound momentarily distracts me. Having broken the spell of the article, I ask myself whether I will return to it immediately, or have a coffee first. Shall I continue reading to find where the article is taking me, or shall I stop and enjoy a drink? I am aware of a kind of tension, of opposing impulses. Then I get up from my chair and make myself a coffee. I know the choice has been made, but I experienced no decision process.

Another way one becomes aware of the outcome of one's decision is by one's feelings about the issue under consideration. Depending on the nature of the choice that one faces, one's feeling of knowing the outcome may come quickly, or there may be an intervening period of uncomfortable uncertainty or indecision. And the feeling of decision may come suddenly or it may be a slow realization. When it comes, the feeling could be of preference for a particular object, or an inclination toward a particular action. One may have thought about an issue long and hard, working through all the various considerations, perhaps even writing them down. Then, at some point, one knows one's “conscious” choice.

When making a choice or decision, the step from awareness of the various options to one's decision is a nonconscious process (Nisbett and Wilson, 1977; Johansson et al., 2005, 2006). In an investigation of the decision process, Petitmengin et al. (2013), enhanced their subjects' recollection of decisions by an elicitation interview method, so as to access their decision processes. Their subjects recovered much of the sequence of events surrounding their decisions, but there was no reference to their reporting all the data manipulations central to a decision. Their reports are much as one observes when taking careful note of events during the course of a decision; experiencing the sensory and emotional information prior to the decision, followed, perhaps after some delay, by an awareness of one's decision (which has been nonconsciously determined). The experience of becoming aware of the outcome of the nonconscious decision process may be what Petitmengin et al. were reporting (p. 665) when they referred to “an internal criterion informing the subject of the fact that his decision has been made (sense of relief, of determination…).” Petitmengin et al. did not report the actual mental process central to their subjects' decisions, and therefore their research results do not contradict the evidence that the core process of making a decision is not experienced; only events before and after this process are accessible to consciousness.

Haggard and Eimer (1999) investigated the relationship between the timing of neural events and the perceived time of choosing to make one of two alternative voluntary actions (whether to move the left or right index finger). They demonstrated that the decision, “left or right” was apparent from the LRP before the perceived intention to move. They wrote (p. 132) that “the LRP is a relatively late event in the physiological chain leading to action,” but it appeared to precede conscious awareness of an intention to move, therefore the decision was made nonconsciously. In a report of functional magnetic resonance imaging studies during decision processes, Soon et al. (2008, p. 545) wrote that “two specific regions in the frontal and parietal cortex of the human brain had considerable information that predicted the outcome of a motor decision the subject had not yet consciously made.” Both these reports support the view that decisions are nonconscious processes.

#### 2.2.4. The control of attention is always nonconscious

There are two forms of attention control: exogenous automatic orienting, and endogenous voluntary control. Orienting is automatic and nonconscious, but voluntary control of attention must also be nonconscious because we have no awareness of choosing what we will attend to Velmans (1991).

Velmans (1991, 2009) wrote that it is logically impossible for consciousness to select what enters itself, unless it is already being experienced. This argument must be valid for the very many occasions when our attention moves to something that was not already in mind, but it is not valid for some of our movements of attention. For example, I am hearing a noise, and I attend to it because I am curious where it is coming from; or I look more closely at an insect on a wall; or my attention moves from one flower to an adjacent one in the garden. I was experiencing these things before I chose to focus on them, so are these movements of attention consciously determined?

First, it is likely that the same mechanisms of selection apply whether or not the object was being perceived prior to the movement of attention, which would suggest they are nonconscious. Second, voluntary control of attention amounts to intentionally choosing which information will be experienced and adjusting one's attention accordingly. In relation to the control of attention (just as with other choices, decisions, and control of action) some aspects of the prior information may be conscious, but the crucial decision and control processes are never observed. Voluntary and involuntary movements of attention are both controlled nonconsciously.

### 2.3. Summary and conclusions

In any intentional action, one never experiences the complete sequence of events from the starting conditions to completing the action. Bowers (1984, p. 249) wrote that “one can introspectively notice and/or recall antecedents of one's behavior but the causal connection linking the determining antecedents and the behavior to be explained is simply not directly accessible to introspection. Rather, the causal link between antecedents and their consequences is provided by an inference, however implicit and invisible.”

There are gaps in every experience of intentional choice, intentional initiation of responses, intentional control of attention or behavior, and in thinking, speaking, problem solving, creativity, and every other action with which consciousness is associated; and in each of these activities the executive mental process is missing from consciousness. However, since we never experience mental processes, we cannot know what it would be like to experience them. One can only know that one does not experience these processes by the fact that a stage in the events is absent, though, in the ordinary course of events, one generally does not notice this. All the “real work” associated with consciousness is actually not conscious; all behavior (and experiences) of conscious organisms are under nonconscious control.

The absence of data processing in consciousness was assumed by Velmans (1991, 2002) and others (Pockett, 2004; Robinson, 2012) to imply that consciousness had no biological function. However, in the words of Smith (1999, p. 426), “being unable to ‘act’… does not, however, imply serving no purpose.” Or as Gomes (2005, p. 78) wrote, “consciousness may usefully be considered to influence the other brain systems that directly cause behavior.” Consciousness could be biologically valuable in a non-executive role associated with the generation of behavior. In fact, the evidence that consciousness does have biological value is strong, and this is detailed in the next section.

## 3. Evidence that consciousness has biological value

An issue that is important when assessing the evidence for consciousness being adaptive, is whether or not consciousness could be considered as functionally separable from the conscious neural structure of which it is a property. This question is important because much of the evidence that consciousness is adaptive depends upon information that is common to consciousness and the conscious neural structure. There have been claims that the conscious neural structure has biological value but consciousness is functionless (Pockett, 2004; Blakemore, 2005; Robinson, 2007). This amounts to a claim that, in terms of its functioning, consciousness is separable from the conscious neural structure. There have also been counter claims that consciousness is integral to the conscious neural structure (for example, Gomes, 2005).

There are reasons to believe that consciousness cannot exist without the conscious neural structure, and that the neural structure by its nature produces consciousness, so one cannot have the neural structure without having consciousness (Loorits, 2014). Therefore, the conscious neural structure and consciousness, as its property, are functionally inseparable, and any evidence that consciousness is adaptive is evidence that both consciousness and the conscious neural structure are adaptive.

### 3.1. The complexity argument that consciousness is adaptive

A number of scientists have commented that consciousness appears to be so complex it must be adaptive (for example, Gray, 1971, 1995; Nichols and Grantham, 2000). Consciousness is certainly complex, incorporating several sensory modalities, each with a number of variants, interrelated with various felt experiences.

When one considers the complexity of visual perception, one appreciates that this aspect of consciousness must have evolved in many stages, sufficient to establish that visual consciousness does have biological value. According to Reber (1992), cerebral achromaticity is rare, but dark–light blindness is much rarer, and Jackson's Principle states that evolutionarily older functions are more robust than more recent functions. This suggests that dark–light experience developed separately and earlier than color experience. Additionally, a system has evolved whereby the colors that one experiences are adjusted so as to give maximum differentiation between objects (Gouras and Zrenner, 1981; Gouras, 1991; Thompson, 1995).

Light–dark contrast, in combination with the color spectrum, as it is employed in consciousness, is a rather complex method for differentiating objects within our visual experience. This almost certainly arose in stages over a significant period of evolutionary time, which could not have occurred without it offering survival advantages. If visual experience provides survival advantages, consciousness, of which visual experience is an integral part, must also provide survival advantages. But, the total complexity of consciousness is much greater because it includes many other interrelated components^8^: auditory experiences of pitch and loudness; tastes and odors representing chemical information; internal awareness of contact with objects; awareness of one's own bodily position and movements; perception of one's emotions and other internal states, and one's feelings about people objects, and events. And each of these is normally appropriately positioned within a three-dimensional array. Consciousness is an extremely complex phenomenon that could not have evolved without having an important biological function.

### 3.2. Evidence from the evolution of accessory systems associated with consciousness

The existence of accessory systems that have evolved in association with consciousness is evidence that consciousness has biological value and can influence behavior:

1. The vision for perception system has evolved in addition to the vision for action system (reviewed in Rossetti and Pisella, 2002; Glover, 2004; Goodale and Milner, 2004; Goodale and Humphrey, 2005; Goodale, 2007; Umiltá, 2007). The fact that a second visual system with different properties has evolved to provide suitable information for conscious perception must mean there is biological value in perceptual systems.
2. “Explicit” or “declarative” memory, associated with consciousness, has evolved in addition to “implicit” or “nondeclarative” memory systems that serve fully nonconscious mechanisms (Graf and Schacter, 1985; Schacter, 1987; Berry, 1993; Zola-Morgan and Squire, 1993; Eichenbaum, 1994; Squire, 1994), and this could only have occurred because consciousness is adaptive.
3. Experimental evidence that nonconscious systems have evolved for extraction of suitable external data for consciousness (McCauley et al., 1980; Marcel, 1983; Groeger, 1984, 1986). This could only have occurred because consciousness has biological value.

### 3.3. Evidence from the correlation between consciousness and actuality

James (1879) wrote that we evolved pleasant feelings toward what is generally good for us and unpleasant feelings toward what is generally bad for us. He said that if consciousness had no effects, we could quite easily have evolved with pleasant feelings toward what harms us and unpleasant feelings toward what is good for us, but we did not, therefore it is likely these feelings, and hence all experiences, are adaptive.

James' argument can be extended from feelings to all of our experiences. When we are about to do something, if it is not a fully automatic response, we are conscious of our intended action. Why would it be necessary to know our intentions unless our experiences have some influence on our behavior? Also, whenever we are actively involved with events, consciousness is fairly well correlated with the facts of the situation. This is ensured by a mechanism for reality monitoring of experiences in our ordinary waking states, whenever that is necessary (Johnson and Raye, 1981). If consciousness had no effects on behavior, it would not matter if our experiences were completely fantastical and had no correlation with reality. But our experiences have evolved so as to represent reality fairly accurately whenever that is necessary, and this is evidence that consciousness can influence behavior, and is adaptive.

### 3.4. Evidence from the special treatment of self-related information in consciousness

In general, there is a very clear separation between self-related information and external sense information in consciousness. Bodily sensations like pain, coldness, hunger or tiredness; emotional and other feelings, like anger, confidence or pleasure; knowledge of our own choices; and awareness of our own physical boundaries, are normally well differentiated from exogenous information.

In normal conditions, self-related information from diverse internal sensors is always perceived as self-related, and always located together. If consciousness had no biological function, it could easily have been otherwise—it would have made no difference in terms of one's survival if self-related information were scattered across one's experience. The fact that the perception of self-related information has evolved so as to be experienced as grouped together, and to have the special quality of personally relating to oneself, is evidence that consciousness has biological value.

### 3.5. Consciousness can directly influence actions

Conscious information can have a dominant influence on responses. We tell others about our experiences, write about our experiences, and think about our experiences, so consciousness must contribute to the generation of these behaviors (for example, Blackmore, 2004; Gomes, 2005). In everyday situations, one is aware of an intention to do something, and then does it. Or our feelings may interrupt our thoughts and alert us to another priority, and we may change what we are doing.

REM atonia—the blocking of messages to major muscles during REM dreams, when reality monitoring is switched off—prevents us acting out our dreams (Hobson, 1988; Jouvert, 1999), and could only have evolved because our experiences can provoke actions.

Persaud and Cowey (2008) reported experiments with a blindsight patient, GW, in which he was asked to report on a stimulus present either in his blind field or his normal field. GW was asked to say “Up” if the stimulus was in a lower quadrant of his visual field, or his blindsight field; or “Down” if it was in an upper quadrant. He correctly reported the opposite location to that of the stimulus in his sighted field, but significantly more often than chance he incorrectly reported the actual quadrant when the stimulus was in his blind field. He was only able to do as instructed, and override his automatic tendency to report the actual quadrant the stimulus was in, when the stimulus was consciously detected.

There is considerable experimental evidence for the dominance of conscious events over automatic action programs (for example, the experimental data in McCauley et al., 1980; Marcel, 1983; Groeger, 1984, 1986; Merikle and Joordens, 1997; Rossetti, 1998; Haggard and Johnson, 2003). In each of these experiments, when non-conscious processes extracted multiple interpretations, the single interpretation that consciousness was able to access had a dominant influence on subjects' responses.

### 3.6. Evidence from the existence and rareness of qualia^9^

Important evidence that consciousness has biological value is provided by the existence of qualia. Consciousness evolved as an array of qualia of various kinds that has the capability to represent visual properties such as relative size, location, movement, shape and texture, and quantitative and qualitative properties of information from other sensory modalities. This contributes to the breadth of conscious information, and thereby to the complexity argument.

If qualia were a property of all neural states, every neural event would be conscious, which is obviously not so. Qualia are a very rare property of neural states; and how they arise is, at present, unknown. The fact that qualia exist but are such a rare phenomenon indicates that they have evolved with special properties for a particular function (see Section 4.2).

### 3.7. Summary of evidence that consciousness has biological value

Consciousness is a function of living organisms, and it is unsurprising that it is adaptive, since most functions of organisms have evolved to enhance their biological fitness. In summary, the evidence that consciousness has biological value is:

1. Consciousness is very complex.
2. Various ancillary systems have evolved in association with consciousness.
3. Whenever one is actively involved with events, one's experiences are representations of them.
4. Self-related information, which is obviously very relevant to survival, is treated differently from all non-self information.
5. Consciousness appears to directly influence behavior.
6. We have consciousness because we have qualia, which are very unusual properties of neural states and appear to have evolved for their ability to convey important information.

We can conclude that consciousness does have biological value, though it includes no mental processes. Therefore, consciousness must have a nonexecutive biological function—a secondary or supporting role to associated neural mechanisms that do have executive functions. In the next section, I demonstrate that consciousness is a changing array of various types of information, and, incidentally, that when we analyze consciousness into its components, we find no processes of any kind.

## 4. Evidence that consciousness is solely information

### 4.1. The components of consciousness are all forms of information

A number of researchers have claimed that consciousness—one's experience from moment to moment—consists of information in various forms (Battista, 1978; Dretske, 1995, 2003; Tye, 1995; Chalmers, 1996; Lycan, 1996; Mangan, 1998; Armstrong, 1999; Smith, 1999). In this section, I discuss the interrelated components of consciousness that together constitute one's experiences. Analysis of the components (listed in **Table 1**) and the way they are structured, demonstrates they are all information and that consciousness is solely information of various kinds in a continuously changing array.

**Table 1:** **The components of conscious experience**.

**Component**	**Example experiences**
External sense experiences	Visual, auditory, olfactory
Felt experiences	
Transitional	Touch, weight, hardness
Physical state	Hunger, pain, proprioception
Emotional	Anger, joy, fear
Mood	Happiness, sadness
Evaluative	Liking, doubt, comprehension
Associated information without qualia	Intentions, identities of things

As noted previously (in Footnote 5), I use the term “information” to mean data or facts, which is a broader meaning than the usage in information theory as reduction in uncertainty. Some examples should help clarify my usage: The fact that I know something is information, and what I know is information. The fact that I am in pain is information, but the pain itself is information about possible bodily damage (Chapman and Nakamura, 1999). The fact that I am angry is information, but my feeling angry is itself information about my own response to events (Schwarz and Clore, 1996). Each of these statements does more than simply reduce uncertainty; it establishes the meaning or context of the information.

*External sense experiences* are information about real, imagined, or remembered external objects or events, represented as colors, shapes, sounds, smells, and so on.

*Transitional feelings* are based upon external sense data detected via sensors in the skin or musculature. Experiences such as contact with surfaces, awareness of surface texture, and awareness of the hardness or weight of an object, are primarily based upon exogenous data, but they are mediated by cutaneous and muscle receptors. These feelings incorporate both exogenous and endogenous information; to varying degrees they convey information about the external situation and information about the associated internal state (Katz, 1925/1989).

*Physical state feelings* represent information about internal physical conditions (Schwarz and Clore, 1996). For example, pain is a representation of bodily disturbance (Tye, 1997); it is normally a “message of peripheral tissue trauma” (Chapman and Nakamura, 1999, p. 392); and hunger informs consciousness of a need or desire for food.

*Emotional feelings* are representations (Damasio, 2001)—information (Schwarz and Clore, 1996)—concerning our state of physical and psychological responding to actual events, or to memories, thoughts, or imaginings (Kleinginna and Kleinginna, 1981; Scherer, 1984; Lazarus, 1991; Izard, 1993).

*Mood feelings* represent emotional states that are not tied to a particular situation, and are less well differentiated than other emotional feelings (Clore and Bar-Anan, 2007; Isbell and Burns, 2007). They inform consciousness concerning one's pre-existing psychological state or response bias (Schwarz, 2002). Therefore, these feelings are information (Schwarz and Clore, 1996).

*Evaluative feelings* are based upon nonconscious evaluations of things, and innate responses or learned associations in relation to them (Zajonc, 1980; Tesser and Martin, 1996; Smith and DeCoster, 2000; Seager, 2002; Slovic et al., 2002; Velmans, 2002; Northoff, 2008). These feelings qualify objects, events, people, ideas, and so on, with regard to their meaning for us, our attitudes to them, or our judgments about them, and result from nonconscious and immediate evaluation processes (Arnold, 1960, 1970; Dixon, 1981; LeDoux, 1987; Lazarus, 1991; Tesser and Martin, 1996; Bargh and Ferguson, 2000). “People's feelings inform them about what they like, want and value” (Clore and Bar-Anan, 2007, p. 14), but also what they understand, distrust, are familiar with, and so on; they are information about one's personal valuation of things (Schwarz and Clore, 1996).

*Information that lacks qualia*, such as the identities of objects or knowledge of one's own intentions, constitutes another component of experience. Evidence for conscious information without associated qualia is discussed in Section 4.3.

We can draw two conclusions about the components of consciousness from these observations:

1. All the components of consciousness are solely information in various forms; consciousness is a changing three-dimensional perceptual array of information. It follows that, since consciousness is solely information but is adaptive, and therefore can influence behavior, it must function as input data to a process, or processes, that determine behavior.
2. Consciousness includes no mental processes; we experience the results of mental processes, we do not experience the actual processes. The only experiences that might superficially seem like processes are transitions from one group of sensations to the next, from one feeling to the next, from one experienced emotion or mood to the next, or from one thought to the next. But each of these is merely a change in the experienced information, which is generated by processes outside of consciousness. These transitions correspond to changing information in conscious neural structures, and result from nonconscious processes. They are analogous to events on a TV screen that merely reflect the changing outputs of unseen electronic processes elsewhere in the TV. Consciousness includes no processes.

### 4.2. Qualia incorporate qualitative and quantitative information into consciousness

Consciousness is information, and the information of consciousness is represented or encoded as qualia; as sounds, colors, feelings, and so on. Because we have a perceptual array of qualia, we are conscious and this is important to us. But (as noted in Section 3.6), qualia are also important because they permit information about various qualitative properties, such as color or texture, and quantitative properties, such as relative size and location, to be incorporated into the information of consciousness. The ability to incorporate these properties is a feature of qualia; and no qualia have evolved to represent data that do not have these properties (Section 4.3). Therefore, the ability to incorporate qualitative and quantitative properties would seem to be the reason that qualia evolved.

### 4.3. Information lacking qualia that is experienced

There appears to be information associated with consciousness, such as the identities or functions of things, and our own intentions, which has no qualitative or quantitative properties, lacks qualia, and has no location in the perceptual array. If I am given an object whose function is unknown to me, and I examine its colors, shape and size, I learn about its qualia. Perhaps I also recognize that it is made of timber and metal. The qualia array gives me clues to the identities of the materials from which it is made, but my recognition of these materials depends upon my past experience and is not a property of the qualia, as such. When someone informs me of the purpose of the object, I learn something new about it, but its qualia remain unchanged. Initially, its purpose is represented in words, but later I know what it is for without putting that knowledge into words. The function of the object and the materials from which it is constructed are knowledge that is additional to what is directly conveyed by the qualia array, and when these facts are well known they cease to be represented by words, so they are no longer represented by qualia of any kind.

The existence of agnosias supports the view that the identities of people and objects are normally associated with consciousness. Agnosias are defects of awareness, failure of certain forms of information to be experienced (Farah, 1992, 2004; Behrmann and Nishimura, 2010). In the associative agnosias, certain information associated with items being experienced, such as the meanings of words or the identities of objects or faces, are not consciously accessible, though there is evidence they are nonconsciously known. Since those of us with normal perceptual systems are able to consciously remember and tell others about the identities of objects or people, these facts are normally associated with consciousness when necessary.

There have been previous reports that some thoughts lack qualia. Siewert (1998) gave examples of what he called “noniconic thinking,” everyday events when he considered his thoughts were neither expressed in pictures nor in words: suddenly realizing he might have missed an appointment; discovering he did not have his house key in its usual pocket, then remembering he had transferred it to his coat pocket; or approaching a green traffic light, and wondering if it was about to change. If thoughts are not expressed in words or pictures; in sounds or images; and if they have no associated feelings of any kind, they lack qualia.

Hurlburt, Heavey and Akhter (Heavey and Hurlburt, 2008; Hurlburt and Akhter, 2008a,b) state there are well-defined experiences, such as unspoken thoughts, wonderings, musings, and unspoken knowledge of where we are, or what we are looking at, that are conscious without qualia. They refer to these experiences as “unsymbolized thinking,” and reported that about one quarter of the randomly sampled experiences of 30 students included unsymbolized thinking.

Qualia incorporate quantitative and qualitative properties of things into consciousness, and that appears to be their function. Unsymbolized thought, such as knowledge of the identities of objects or people, one's location, what one is doing, where one is going, or one's intentions, solutions to problems, and so on, often have no associated quantitative and qualitative properties, and no qualia. One experiences such information by “just knowing it,” because it has none of the properties that information represented by qualia have. Various unsymbolized thoughts may be experienced, depending on what one is attending to, in much the same way as, for example, various sensory information may be experienced, depending on what one is attending to.

## 5. Evidence that consciousness is the input data to a flexible response mechanism

Consciousness is information, it is adaptive, and it is associated with intentional behavior. In this section I present evidence that the biological function of consciousness is input data to a mechanism that generates flexible, intentional responses.

### 5.1. Consciousness is associated with a flexible response mechanism

A list of various consciousness-related mental activities is provided in **Table 2**. Inevitably, there are some overlaps between the listed categories, and not every activity may be included, but they cover the great majority of activities associated with consciousness, sufficient to provide a basis for investigating the biological role of consciousness. When we examine this range of intentional behaviors associated with consciousness, we find that consciousness is primarily associated with flexibility of behavior.

**Table 2:** **Mental activities associated with consciousness**.

**Function**	**Category of mental event**
Responding to current situations	Interacting with people
	Attending to a sudden or unexpected event
	Close attention to a task
	Alertness in unusual, interesting, or unpredictable situations
	Observing events as they occur
	Initiating an action that is contrary to habit
	Learning a skill
Preparing for expected future actions	Mentally processing instructions
	Remembering or reviewing events
	Thinking through an expected event, or preparing actions by mental rehearsal
	Choices and decisions
Acquiring background to possible future actions	Gaining special or general knowledge
	Thinking about problems for creative resolution over time
	Abstract thought and logical reasoning
	Metacognition
Anomalous processes	Mind wandering
	Dreams during sleep

The last two categories in **Table 2**, mind wandering and dreams during sleep, are anomalous because they do not have the volitional or controlled quality of the other activities in the table, and are generally disconnected from any definite tasks. The anomalous processes have no obvious behavior-related function, though it has been reported that various functions related to preparation for future adaptive behavior are associated with dreaming (Hobson, 2010) and passive mental states, such as mind wandering (Greicius et al., 2003; Buckner and Vincent, 2007). At this stage, the anomalous processes seem unlikely to contribute to our understanding the biological role of consciousness.

All of the activities in **Table 2**, apart from the anomalous processes, are associated with generation of, or preparation for, nonautomatic behaviors to deal with current, expected, or possible future situations. The situations may be internal or external physical conditions, or social conditions, so the actions could be related to personal safety, social status, homeostasis, or other considerations. These activities are all relatively flexible compared to the stimulus-response structure of automatic actions such as the orienting reflex or looming reflex (Schiff, 1965; Ball and Tronick, 1971), fixed action patterns (for example, Raven and Johnson, 1992), unintended motor mimicry (Bavelas et al., 1986; Chartrand and Bargh, 1999), classically or instrumentally conditioned responses, and learned behavior that has become automatized. Consciousness is associated with relatively flexible responding (Baars, 1988), that is, with a flexible response mechanism, or possibly a combination of such mechanisms (the FRM).

The FRM selects or devises responses to current situations, causes the automatic initiation and control of behavior (Bargh, 1992), prepares for possible future events, solves problems, and makes choices. Each of these must be achieved by integration and manipulation of relevant data. The FRM must arrive at nonautomatic solutions to problems and determine behavior by information processing of some kind, and therefore must consist of a processor operating with relevant data.

### 5.2. The flexible response mechanism operates with selected information

Automatic programs determine most of our responses, and initiate and control all of our actions (Bargh, 1992; Bargh and Chartrand, 1999). The FRM is an alternative system for determining behavior that functions quite differently from automatic programs.

An example of the operation of automatic and intentional systems is the two modes of attention control: automatic detection and orienting, and controlled search (Posner and Snyder, 1975; Shiffrin and Schneider, 1977; Posner, 1980; Norman and Shallice, 1986). A second example of the operation of these systems is the two processes that contribute to reasoning: automatic, intuitive or associative processes; and deliberative or controlled processes (Sloman, 1996; Bargh and Ferguson, 2000; Smith and DeCoster, 2000; Stanovich and West, 2000; Kahneman and Frederick, 2002; Evans, 2003; Evans and Stanovich, 2013). The duality of mechanisms for attention control and reasoning are aspects of the broader duality of mechanisms for generating all our behavior: automatic action programs and the FRM.

As noted in Section 5.1, automatic action programs include innate responses, conditioned responses, and action sequences that were once intentional but have become automatized, and each of these is released by a specific stimulus. Thus, when driving, if a child runs into the road, one's foot hits the brake automatically and very quickly; the automatized driving program ignores all other information, and responds to the external sense data about the emergency. The same principle applies, though less obviously, as we automatically and continuously respond to sense data indicating some more minor adjustments to our driving are needed. In general, automatic action programs, whether innate or learned, respond very rapidly to a relevant stimulus and ignore all other information (Bargh, 1992; Kihlstrom, 1993), and this involves minimal processing of the sense data.

The flexible response mechanism operates differently from automatic programs; it manipulates a selection of relevant information in search of an appropriate response to a particular event or problem. The input to the FRM has to include all essential information so as to maximize the possibility of an optimal response, but must exclude or inhibit information that is irrelevant to the task (Tipper, 1985; Allport, 1987; Tipper et al., 1994; Wühr and Frings, 2008). Exclusion of irrelevant information minimizes unproductive information manipulations that might increase the time taken to arrive at a response, or cause the FRM to switch to a different task. The need to control its input data means the FRM must employ an information selection system of some kind. The operations of the FRM use the selected information, and are generally isolated from all other information.

The suggestion that there is a mechanism associated with the FRM that selects its input data is supported by the work of Leopold and Logothetis (1999). In their review of research on multistable perception, they found evidence of “direct intervention in the processing of the sensory input by brain structures associated with planning and motor programming” (p. 254), and concluded that the brain areas involved are “those that ultimately use and act upon the perceptual representations” (p. 261). A more recent review of multistable perception by Sterzer et al. (2009, p. 317) agreed; they wrote that “high-level frontoparietal processes continuously re-evaluate the current interpretation of the sensory input and initiate changes in subjective perception.” Subjectively, perceptual switches during multistable perception appear to be automatic, and one might not expect that systems associated with planning and motor programming, which use and act upon the perceptual representations, would be involved in sensory data selection or interpretation, were it not for the fact that these operations are necessary for the FRM, which logically must have an associated mechanism for selecting its input data. Therefore, these reviews provide some support for the view that consciousness is the data input to the FRM.

In concluding his review of studies of attentional responses to visual data, Theeuwes (2010, p. 97) wrote, “during the first sweep of information through the brain (<150 ms) visual selection is completely stimulus-driven. Initial selection is based on the salience of objects present in the visual field…. Only later in time (>150 ms)… volitional control based on expectancy and goal set will bias visual selection in a top–down manner.” The sequence and timing Theeuwes reported are as would be expected on the basis that a mechanism associated with the FRM selects data for attention but, because the FRM operates by manipulating relevant data, it is slower than automatically triggered responses.

### 5.3. The input data requirements of the flexible response mechanism correspond to the properties of consciousness

The flexible response mechanism requires a restricted data input, and consciousness, which is closely associated with operations of the FRM, is a limited information flow. This suggests that consciousness could be the input data to the FRM. Support for this view is provided by the fact that the properties of consciousness correspond to the necessary properties of the input data to the FRM:

1. In order to select or devise responses to events, the FRM requires access to relevant exogenous and endogenous data. Consciousness incorporates these as sights, sounds, etc., feelings of various kinds, and unsymbolized thoughts.
2. The FRM requires knowledge of various qualitative and quantitative properties of things, such as size, shape and location; and the qualia array of consciousness has apparently evolved to meet this need (Section 4.2).
3. The FRM requires input data that is a restricted representation of events, because information that is irrelevant to its computations has to be excluded. Any actions following on from the work of the FRM are controlled by sensorimotor systems specialized for those actions, which employ accurate and continuously updated data for action (Rossetti and Pisella, 2002; Goodale and Milner, 2004; Goodale and Humphrey, 2005; Goodale, 2007; Umiltá, 2007). Consciousness is an incomplete representation of events, as is demonstrated by inattentional blindness (Neisser, 1979; Mack and Rock, 1998; Simons and Chabris, 1999). The fact that our experiences are incomplete representations of reality is sometimes noticeable in ordinary life.
4. Some operations of the FRM require information to be held longer than the brief period that data for action are held. In order to make a complex choice or decision, the input data may need to be retained for some time whilst the FRM processes the options and arrives at an outcome. And, when an intended action is delayed, when an action extends over time, or when there is an intended sequence of actions, the input to the FRM may need to be held for an extended period to ensure the action is completed appropriately. Complex decisions and complex intentional actions may require that relevant data be held much longer than the very brief period that data for action are held, and this is a characteristic of consciousness (Darwin and Turvey, 1972; Tiitinen et al., 1994; Damasio, 1995; Rossetti, 1998; Dehaene and Naccache, 2001).
5. Bridgeman et al. (1997, p. 468) found that the “cognitive map,” that is visual consciousness, “can achieve great sensitivity to small motions or translations of objects in the visual world by using relative motion or relative position as a cue.” By comparison, data driving visually guided action “does not have the resolution and sensitivity to fine-grained spatial relationships that the cognitive map has.” Input data allowing increased sensitivity to movement would be an asset to the FRM, in that it could permit enhanced responding in situations of slowly emerging danger or opportunity, in unusual or unpredictable situations, or in some social interactions.
6. The data input to the FRM needs to have a variable correlation with current reality. When responding to a current external situation, the input information has to be closely aligned with the actual events, but when the FRM is reviewing past events, planning future actions, or solving a problem, the required information may have little or no correspondence with current external reality. Consciousness can be information about one's current situation, or it can be information about something quite different that is receiving one's attention.
7. Input to the FRM needs to be primarily context-related unlike data for action, which are self-related. For example, planning future action requires that the context be specified. When driving on an unfamiliar route I need to know the contextual features of a particular side road that I must take to reach my destination. However, at the road junction, I will need to make movements that take into account external factors but are determined in relation to the relevant parts of my own body; the data for action system must precisely determine my limb movements to control the vehicle. Data for action are instructions to automatic programs that move parts of the body, whereas input to the FRM needs to be a representation of the event that includes the context of the perceiver (Rossetti and Pisella, 2002; Goodale and Milner, 2004; Milner and Goodale, 2008). Consciousness has sensory data in context as would be expected of the input to the FRM.

Thus, there is a correlation between the properties of consciousness and the properties required of input data that will allow the FRM to compute responses to a range of actual or expected situations. For this situation to have arisen, mechanisms that determine the various properties of consciousness and of the FRM (and its data selection mechanism), must have evolved in a complementary manner over very long evolutionary time. This could only have occurred because consciousness is the data input to the FRM.

### 5.4. Information missing from consciousness may adversely affect the output of the flexible response mechanism

Whenever any important information relevant to the computations of the FRM is missing from consciousness, the output from the FRM tends to be adversely affected:

1. People with blindsight do not spontaneously respond to data in their blindfield, even though they have nonconscious knowledge of it (Marcel, 1986; Weiskrantz, 1997; Dietrich, 2007; Persaud and Cowey, 2008). Blindsight patients lack visual consciousness in their blindfield, and therefore lack the necessary inputs to the FRM for making spontaneous intentional responses. The blindsight patient who worked with Persaud and Cowey (2008) was unable to follow instructions in relation to data in his blindfield. Analogously, “Dee,” in Goodale and Milner (2004), who has visual agnosia, cannot state the orientation of a slot but she can post a letter through it, and cannot state the shapes of obstacles but can walk up a rough track. Intentional action, in the form of responses to questions, is not possible for her because the necessary visual properties of objects are missing from consciousness, though they are nonconsciously known and acted upon.
2. Damasio (1995) reported that patients with damage to brain regions involved in the generation of emotional and other feelings consistently exhibit dysfunctional reasoning, decision-making and behavior. This would be expected if consciousness is input to the FRM, because people who lack emotional and other feelings lack the nonconscious evaluations these feelings represent, which are sometimes needed to get the best outcomes from complex or difficult decisions (Clore et al., 2001; Clore and Huntsinger, 2007).
3. If one intends a non-habitual action, one is only able to act on one's intention if it is remembered, if it is experienced, at the appropriate time. And, if one begins an intended non-habitual sequence of actions but starts thinking about something else part way through, one may end up completing an unintended habitual action sequence if part of the non-habitual action sequence coincides with it (Reason, 1979; Norman, 1981). Without the necessary input data at each stage, the FRM may not keep the action sequence on track.

The adverse effect on the output of the FRM when significant information is missing from consciousness is further evidence that consciousness is input data to the FRM.

### 5.5. Consciousness is input to the flexible response mechanism

In summary, consciousness has biological value and therefore must influence behavior. But consciousness is solely information in various forms, and, as such, can only influence behavior if it is input to processes that determine behavior. Evidence supporting the proposition that consciousness is the input data to the FRM is provided by the following facts:

1. Consciousness is associated with actions of the FRM.
2. To function effectively, the FRM requires a selected data input, and the properties of consciousness correspond to the requirements for the input data to the FRM.
3. There is experimental evidence that experienced information influences the outputs of the FRM.
4. When important information relevant to the task of the FRM is missing from consciousness the FRM tends to malfunction in some way.
5. Our subjective experience is that when we are attending to a novel or interesting event, paying close attention to a task, interacting with people, learning a skill, or thinking about a problem or an expected event, our perceptions are knowledge about events that activates mental processes which devise, prepare, initiate, adjust, or control our actions, or determine the direction of our thoughts.

However, it is common experience that consciousness can be stages in processing by the FRM, such as when one is thinking through a problem or mentally rehearsing an expected future event. A stage in processing is both output from one stage and input to the next stage, but the functional role of consciousness can only be as input to further processing. One sometimes perceives that consciousness is the output from decisions, planning, creative processes, or logical thought, but these are probably always inputs to further processing, such as thoughts, determining present actions or intended future action, telling other people, or committing to memory for possible future use. Consciousness is inevitably output from some process of data selection or manipulation, but its biological function is the data input to subsequent processes of data manipulation, determining action, interaction with people, and so on, and it would appear that is why it evolved.

## 6. Central to the theory are three claims that may be testable

This theory makes three claims that may prove to be testable, and if any of these claims were shown to be wrong, the theory, in its present form, would have been disproved.

First, the theory states that consciousness is the data input to a nonconscious mechanism, or combination of mechanisms, (the FRM) which determines every aspect of one's life that is not determined by an automatic program or mechanism. (In some circumstances, conscious information may become input data to automatic programs. It is also possible that nonconscious information may influence the processes of the FRM directly, and not just via its effects on consciousness, but I have been unable to find any evidence of this).

Second, consciousness functions solely as input data; every component of consciousness is information in some form, and no part of one's experience is ever not information.

Third, all mental processing is nonconscious; one does not experience any mental process. Therefore, one cannot have direct knowledge of the reasons for one's actions, intentions, choices, or decisions; or of how one's thoughts or feelings originated. According to the theory, direct knowledge of these is not possible because consciousness has no access to the processes that determine them. One can only know the reason for any of one's actions, intentions, choices, decisions, feelings and thoughts, by indirect means such as an inference or a guess.

## 7. Conclusions

The behavior of all organisms is principally determined by automatic response programs: innate responses such as orienting and fixed action patterns, classical and operant conditioning, and other learned behaviors. Each of these is automatically released, or triggered, by a predetermined type of stimulus.

However, organisms that possess only automatic responses may sometimes have no response to match a situation that confronts them, and some kind of best choice response, no response, or a random response is used, any of which could result in a missed opportunity or a risk to the organism. Because of this vulnerability, a flexible response mechanism (FRM), which may perhaps be a combination of mechanisms, has evolved to generate responses to novel situations, and consciousness is a component of this mechanism.

The FRM manipulates relevant incoming data, in conjunction with previous learning, in search of an appropriate response to a situation. This can result in suitable behavior being generated, which, if it is repeated, may eventually become automatized (Shiffrin and Schneider, 1977; Bargh and Chartrand, 1999).

The FRM needs access to all information relevant to the situation to which it is seeking to respond, but any information that is unnecessary or irrelevant to the task needs to be excluded from its input because it could increase the complexity of operations and the time taken to achieve an outcome. Hence, a data selection system appears to have evolved, in conjunction with the FRM, which permits access to necessary information and inhibits access to irrelevant information. The operations of the FRM are generally isolated from information that has not been selected.

In order for the FRM to function optimally, its input data need to include qualitative and quantitative information, such as the size, shape, location, and movement of objects. Qualia as a perceptual array allow such information to be incorporated into the input data, and they make the input data conscious. Other forms of information (unsymbolized thoughts) that are relevant to the task of the FRM but do not have quantitative or qualitative properties, such as knowledge of the identities or functions of objects, or of one's own intentions, are also included in the input data, but without qualia.

The FRM utilizes consciousness as its input data and generally cannot access other information, therefore relevant endogenous information has to be included in consciousness. Endogenous information may be experienced as felt sensations, emotions, moods, and evaluative feelings, such as liking or disbelief. And when endogenous or exogenous information is particularly important, a measure of its importance is included in consciousness as experienced emotionality or affect strength.

One's ongoing experience is often an intermediate stage of processing or an output from processing in the FRM, but it is possible that in every case these are inputs to further processing or to other tasks of the FRM. When the FRM has no definite task, it continues to be active with dreams, fantasies and mind wandering. Whether these operations have biological value, and in what way consciousness might contribute, is not yet clear.

The theory that consciousness is input data to a mechanism for generating nonautomatic responses, leads to explanations for the following central features of consciousness, experimental observations, and everyday properties of consciousness:

1. The existence of consciousness as a qualia array (Section 4.2) plus unsymbolized thoughts (Section 4.3).
2. The representation of endogenous information as experienced sensations and feelings of various types (Section 4.1).
3. The experience of the importance of events as affect strength or emotionality.
4. Consciousness is an incomplete representation because irrelevant information is excluded from the input to the FRM, and this explains experimental observations of inattentional blindness (Section 5.3).
5. Distractions interfere with efforts to solve a difficult problem because they represent information that is irrelevant to the problem, which may slow the operations of the FRM, or provoke a task switch away from the topic it is working on (Section 5.2).
6. The FRM is generally isolated from nonconscious information, leading to observations that blindsight patients do not intentionally and spontaneously initiate responses to events in their blind field; and to the everyday observation that an intended counter-habitual action is only possible when the intention is in mind (Section 5.4).
7. If one is distracted during a non-habitual action sequence, one's actions may be captured by a habitual action sequence and completed in an unintended way. This can be understood in terms of the FRM needing appropriate input to continue the intentional action whenever part of the new sequence coincides with an established habitual action (Section 5.4).
8. Damage to brain regions involved in the generation of emotional and other feelings is consistently associated with dysfunctional reasoning, decision-making and behavior. Because these feelings are missing, the input to the FRM is incomplete, and dysfunctional responses are likely when information that would have been represented by the missing feelings is necessary for an appropriate outcome (Section 5.4).

Prior to the first appearance of the FRM in organisms, all of their systems for selecting and initiating behavior were entirely nonconscious, automatic programs (Reber, 1992; Evans, 2008). The FRM is also a functionally nonconscious response system, but with its information input mostly in the form of an array of qualia that provides its possessors with experiences.

The material presented here constitutes the essential elements of a theory that consciousness is the data input to a flexible response mechanism, but there remain many unanswered questions: What is the relationship of the FRM with automatic response programs and with action control systems; what are the mechanics of information selection and data manipulation in the FRM; is the FRM a unitary system or a number of “flexible modules” for decisions, oversight of intentional actions, planning, problem solving, and so on, which separately access consciousness as their input; and why did an array of qualia evolve as the method for qualitative and quantitative data entry to the processes of the FRM?

One would like to know how the FRM operates in non-human species, how its presence can be detected with confidence in non-human species, and whether the FRM has independently evolved in different taxonomic groups (such as cephalopods), which one might expect, since the FRM is a valuable asset that enhances biological fitness. If the FRM has independently evolved in different taxonomic groups, it is possible, though perhaps unlikely, that the problem of representing quantitative and qualitative data may have been solved in ways that do not require a qualia array and do not confer consciousness on their possessors. When we better understand how the FRM evolved and how it functions, we may have more insight into such matters.

It is my hope that this research will open up new directions in the study of the minds of humans and other animals, and that the FRM theory of consciousness may be of use to some of the many researchers who seek to alleviate mental disability and suffering.

### Conflict of interest statement

The author declares that the research was conducted in the absence of any commercial or financial relationships that could be construed as a potential conflict of interest.

## Footnotes

^1^*Consciousness* is used in the sense of phenomenal consciousness (Block, 1995); one's ongoing experience.

^2^*Conscious neural structure:* Consciousness is a property of neurons in a certain state or a certain structure, and I refer to this neural structure as a “conscious neural structure.”

^3^*Mental* refers to events or information in the mind; and *mind* refers to what thinks, feels, reasons, and so on, and which is the seat of consciousness. Mind includes consciousness, the conscious neural structure of which consciousness is a property, and nonconscious mechanisms that manipulate information in reasoning, planning, and determining behavior. I assume all these components of mind are physical properties of events in the brain, based on the view, standard across the natural sciences, that all phenomena are physical phenomena.

^4^*Process* is used in the sense of an action, or actions, that lead to a change in something. Any change in what one experiences is a result of mental processes, that is, the action of manipulating information in the brain.

^5^*Information*, as used here, is data, or facts; it may include data or facts from physical and other measurements or observations, or from any other source, and the results of calculations, inferences, and mental processes, or outputs from other data processing, and their conscious or nonconscious representations. (This usage of “information” is different from the information theory usage as reduction in uncertainty, which is the quantification of a property of information, as I use the term. “Information” as I use it is a broader term; the difference could be expressed by saying that as used here, information also has meaning, or each piece of information establishes a context within which it may reduce uncertainty).

^6^*Nonconscious* refers to information or events in the brain that are not being experienced, some of which are experienceable, some not; it is synonymous with one meaning of “unconscious.”

^7^*Data*, as used here, are primary information, such as information from the external senses; or are information that is input to, being processed in, or output from a mechanism for manipulating, or processing, information.

^8^I use the expression *components of consciousness* rather than the commonly used *contents of consciousness* for two reasons. First, components suggests parts that contribute to some structure or whole, which seems appropriate in a metaphor for consciousness, whereas contents does not imply any connection or structure between the parts. Second, contents implies the existence of some sort of container, which must be “empty consciousness.” Consciousness is one's experiences, but empty consciousness would be consciousness with no experiences, which is a contradiction in terms. Therefore, it would seem that contents of consciousness is not a good metaphor, and components of consciousness is preferable.

^9^*Qualia* are the elements that together constitute ones experiences: sensory experiences, such as colors or sounds; bodily sensations, such as pain, hotness, or thirst; experienced emotions; felt moods; and evaluative feelings, such as belief or liking.
